# The selective cytotoxicity of silver thiosulfate, a silver complex, on MCF-7 breast cancer cells through ROS-induced cell death

**DOI:** 10.1007/s43440-021-00260-0

**Published:** 2021-04-17

**Authors:** Akira Ota, Masataka Tajima, Kazunori Mori, Erika Sugiyama, Vilasinee Hirunpanich Sato, Hitoshi Sato

**Affiliations:** 1 Division of Pharmacokinetics and Pharmacodynamics, Department of Pharmacology, Toxicology and Therapeutics, School of Pharmacy, Showa University, 1-5-8 Hatanodai, Shinagawa-ku, Tokyo, 142-8555 Japan; 2 Division of Cancer Cell Biology, Department of Pharmaceutical Sciences, School of Pharmacy, Showa University, 1-5-8 Hatanodai, Shinagawa-ku, Tokyo, 142-8555 Japan; 3 Department of Pharmacology, Faculty of Pharmacy, Mahidol University, 447 Sri-Ayuthaya Road, Rajathevi, Bangkok, 10400 Thailand

**Keywords:** Silver(I) complexes, Silver thiosulfate, Reactive oxygen species (ROS), Anticancer effects, Cancer cell selectivity, MCF-7 breast cancer cells

## Abstract

**Background:**

Silver is a transition metal that is known to be less toxic than platinum. However, only few studies have reported the anticancer effects of some silver complexes and their possibility as an alternative to platinum complex. This study investigated the anticancer effects of the silver thiosulfate complex (STS), [Ag(S_2_O_3_)_2_]^3−^, consisting of silver and sodium thiosulfate.

**Methods:**

In vitro cytotoxic activity of STS was investigated comparatively in human cancer cell lines (K562 and MCF-7) and normal human cells (mesenchymal stem cells and mammary epithelial cells). For its anticancer effects, cell cycle, mode of cell death, morphological changes, and accumulation of intracellular ROS and GSH were evaluated in MCF-7 to provide mechanistic insights.

**Results:**

STS showed a concentration-dependent cytotoxicity in MCF-7 cell, which was abolished by pretreatment with N-acetylcysteine, suggesting ROS accumulation by STS. Moreover, STS caused cell cycle arrest at the G1 phase, decrease in the GSH levels, and morphological changes in MCF-7. Direct measurement of ROS demonstrated the elevation of intracellular ROS accumulation in cancer cells treated with STS; however, neither cytotoxicity nor ROS accumulation was observed in normal human cells.

**Conclusion:**

The results obtained here are the first evidence to show that STS exhibited an anticancer activity through ROS-induced mechanisms, and that its cytotoxicity is highly selective to cancer cells. The results of the present study warrant further investigation on the detailed mechanism of STS actions, as well as its in vivo effectiveness and safety for clinical application.

## Introduction

Platinum complex-based anticancer agents, such as cisplatin, carboplatin, and oxaliplatin, are currently used for many cancer patients. These platinum complexes damage the DNA of cancer cells but cause various severe adverse effects because they exert their action not only on cancer cells but also on normal cells [1]. Therefore, in recent years, some attempts have been made to develop new metal-based anticancer complexes that cause fewer side effects and can be used as alternatives to platinum complexes. In this regard, studies have recently focused on non-platinum transition metals, such as ruthenium, rhodium, iridium, gallium, titanium, gold, and silver [2]. Because silver has low cytotoxicity to normal human cells, silver complexes could be important candidates for anticancer drugs [3]. Organic compounds containing silver have been employed as infection prevention materials, such as wound-covering agents, surgical instruments, and implants [4, 5]. The use of silver-containing biological agents in the field of infectious diseases is presently limited to silver sulfadiazine, a topical drug used for burns [6]. Silver also exerts antibacterial activity and is therefore widely used in daily needs and products, such as antiperspirants [7].

There are a few reports on the anticancer activity of silver complexes. For example, silver(I) methoxyphenyl phosphine [8], silver with *N*-heterocyclic carbene (NHC) coordinated [9], and cyanobenzoyl-NHC silver complex [10] are cytotoxic to cancer cells in vitro. In particular, [Ag(S_2_CN(C_2_H_5_)_2_)_6_] has an antiproliferative effect in mice bearing A549 lung cancer cells [11]. Silver thiosulfate (STS), [Ag(S_2_O_3_)_2_]^3−^, is a silver complex synthesized from silver and sodium thiosulfate. Among the silver complexes, STS has long been known to have antibacterial activity [12]. However, there are no reports on the anticancer effects of this complex.

Sodium thiosulfate is an antioxidant and is used as an antidote for cyanide poisoning as a pharmaceutical product. It is used at a high dose of 12 g per dose in clinical practice and is considered to be a relatively safe drug [13]. Sodium thiosulfate exhibits a renal protective effect by binding to cisplatin and forming an inactive complex, and it provides effective protection against cisplatin-induced hearing loss in children [14, 15]. Thus, sodium thiosulfate has beneficial effects in treating addictions and reducing chemotherapy-induced adverse effects. Therefore, this study aimed to investigate the in vitro anticancer activity of STS. For this purpose, cell viability tests were conducted using established cancer cells and normal cells to examine cell cytotoxicity caused by STS and its selectivity. Consecutively, the breast cancer cell line MCF-7 was used to study the effect of STS on cell cycle, cell death, cell morphology, intracellular reactive oxygen species (ROS) levels and glutathione (GSH) levels, to clarify the mechanism underlying the anticancer effects of STS.

## Materials and methods

### Cell lines

K562 cells were obtained from RIKEN BioResource Research Center (Ibaraki, JAPAN) and grown in Roswell park memorial institute-1640 (RPMI-1640) (Sigma-Aldrich, St. Louis, MO, USA) medium containing 10% heat-inactivated fetal bovine serum (FBS; Biowest, Nuaillé, France). MCF-7 cells were obtained from the American Type Culture Collection (Manassas, VA, USA) and grown in Dulbecco’s modified Eagle’s medium (DMEM: FUJIFILM Wako Pure Chemical Corporation, Osaka, Japan) containing 10% heat-inactivated FBS. Human mesenchymal stem cells (HMSC) from the bone marrow were purchased from LONZA (#PT-2501, Walkersville, MD, USA) and grown in MSCGM SingleQuots®, purchased from LONZA. Immortalized human mammary epithelial cells (HMEC) were obtained as a kind gift from Dr. Motoko Shibanuma (School of Pharmacy, Showa University) and maintained in MCDB170 (US Biological, Salem, MA, USA)-based medium as described previously [16].

### Synthesis of STS (Na_3_[Ag(S_2_O_3_)_2_])

Silver nitrate (0.4 g) was first dissolved in 20 mL of ultrapure water, and NaCl (0.14 g) was then added to the silver nitrate solution, which resulted in a white precipitate of AgCl. The AgCl precipitate was washed thrice with ultrapure water. Sodium thiosulfate (1.6 g) and ultrapure water were added to the AgCl precipitate to dissolve it completely using ultrasonic waves. Finally, ultrapure water was added to make up the total volume to 200 mL.

### MTS assays

Cells were seeded in a 96-well plate (0.5 × 10^4^ cells/well). After 24 h of incubation, an apoptosis inhibitor (Z-VAD-FMK (25 µM) (Selleckchem, Houston, TX, USA)), a ferroptosis inhibitor (ferrostatin-1 (1 µM) (Sigma-Aldrich)), and an antioxidant *N*-acetylcysteine (NAC (1 mM)) were added to the cells, and the plate was incubated for 1 h. Then, we added aqueous solutions of STS or sodium thiosulfate (FUJIFILM Wako Pure Chemical Corporation) at different concentrations. After incubation for 48 h, 20 µL of MTS solution [3-(4,5-dimethylthiazol-2-yl)-5-(3-carboxymethoxyphenyl)-2-(4-sulfophenyl)-2H-tetrazolium, inner salt] (Promega, Madison, WI, USA) was added to each well, and the plate containing the cells was incubated. Cell viability was measured as being directly correlated to the determined values at absorbance of 620 and 460 nm (BioRad, iMARK™, Hercules, CA, USA).

### Measurement of cell death

MCF-7 cells were seeded in a 6-well plate (15 × 10^4^ cells/well). After incubation for 24 h, an aqueous solution containing 24 µM of STS was added, and the plate was incubated for 30 min. After incubation, the cells were harvested with TrypLE Express (Thermo Fisher Scientific, Waltham, MA) and washed with Dulbecco’s phosphate-buffered saline (DPBS, FUJIFILM Wako Pure Chemical Corporation). After washing, the cells were suspended again in DPBS containing 10 µg/mL propidium iodide (PI: Sigma-Aldrich). Cell death was analyzed using MoFlo Astrios EQ (Beckman Coulter, Inc., Fullerton, CA, USA) [PI (ex. 488 nm, em. 644/22 nm)].

### Evaluation of cell cycle

MCF-7 cells were seeded into a 6-well plate (15 × 10^4^ cells/well) and incubated for 24 h. The cells were treated with STS (12 and 24 µM) and incubated for 24 h. After incubation, the cells were harvested using TrypLE Express and washed twice with DPBS. The cells were fixed in 70% ethanol overnight at 4 °C. Ethanol was removed, and the cells were washed twice with DPBS. The cells were then resuspended in DPBS containing 0.25 mg/mL RNase A (Sigma) and 10 µg/mL of PI. The samples were incubated in the dark for 30 min. Before the measurement, the samples were filtered through a 40-µm nylon mesh. Cell fluorescence was measured using S3e™ Cell Sorter (Bio-Rad) [PI (ex. 488 nm, em. 620/29 nm)]. The values were obtained from at least 10,000 cells, and data were expressed as mean ± SD.

### Immunocytochemistry

MCF-7 cells grown on coverslip were incubated with STS (24 µM) for 48 h. Cells were fixed with 4% paraformaldehyde (FUJIFILM Wako Pure Chemical Corporation) and permeabilized with 0.2% Triton X-100 (FUJIFILM Wako Pure Chemical Corporation). After blocking with 3% bovine serum albumin (BSA: Sigma-Aldrich) in PBS, cells were incubated for 1 h with primary antibodies against Proliferating Cell Nuclear Antigen (PCNA) and cleaved caspase-3 (Cell Signaling Technology, Danvers, MA, USA) and wash with 0.1% Tween 20 in PBS. Then, cells were treated with Alexa Fluor 488- and 568-conjugated goat anti-mouse and rabbit secondary antibody (Thermo Fisher Scientific). The nuclei were stained with DAPI (FUJIFILM Wako Pure Chemical Corporation). After washing, laser scanning confocal microscopy was performed using FV10i-LIV confocal microscope (Olympus Corporation, Tokyo, Japan). To quantify PCNA expression, fluorescence intensity of nuclear PCNA was quantified using ImageJ software (National Institutes of Health, Bethesda, MD, USA). Staurosporine (FUJIFILM Wako Pure Chemical Corporation) was used as the positive control for apoptosis.

### Measurement of intracellular ROS levels

Cells were seeded into a 6-well plate (15 × 10^4^ cells/well) and incubated for 24 h. After further incubation with 1 mM NAC for 1 h at 37 ℃, the cells were treated with STS (12 µM and 24 µM) for 30 min. Cells harvested with TrypLE Express and washed with DPBS. After washing, the cells were suspended in DPBS containing 10 µg/mL PI and incubated with 10 µM 2′,7′-dichlorodihydrofluorescein diacetate (H2DCFDA) (Thermo Fisher Scientific) for 10 min in the dark. After culturing, ROS levels were analyzed using MoFlo Astrios EQ (Beckman Coulter, Inc.) [H2DCFDA (ex. 488 nm, em. 513/26 nm), PI (ex. 488 nm, em. 644/22 nm)]. Tert-butyl hydroperoxide (tBHP) (FUJIFILM Wako Pure Chemical Corporation) was used as the positive control for ROS accumulation. The values were obtained from at least 10,000 cells, and data were presented as mean ± SD. Debris and dead cells were excluded by forward scatter, side scatter, and PI gating.

### Measurement of intracellular glutathione levels

Cells were incubated for 48 h with STS in 96-well plates. Intracellular glutathione (GSH) levels were quantified using GSH-Glo™ glutathione assay (Promega) according to the instructions. Luminescent signals from Ultra-Glo™ Luciferase were measured using a Varioskan LUX microplate reader (Thermo Fisher Scientific), and the values were expressed as ratios relative to the controls after normalizing the cell number.

### Statistical analyses

Data were analyzed using JMP®pro15 (SAS Institute Inc., Cary, NC, USA). Each in vitro assay was performed at least three times. The data of each assay are expressed as the mean ± SD. Differences between groups were assessed using one-way analysis of variance (ANOVA), followed by Tukey’s post hoc test. The difference in the presence and absence of NAC pretreatment was analyzed using two-way ANOVA followed by Tukey’s post hoc test. Student’s *t* test was used to analyze the significant differences between two groups. Statistical significance was evaluated at *p < 0.05, **p < 0.01, or ***p < 0.001 vs. the control group or the non-treatment group.

## Results

### Cell viability assays

**Fig. 1:**
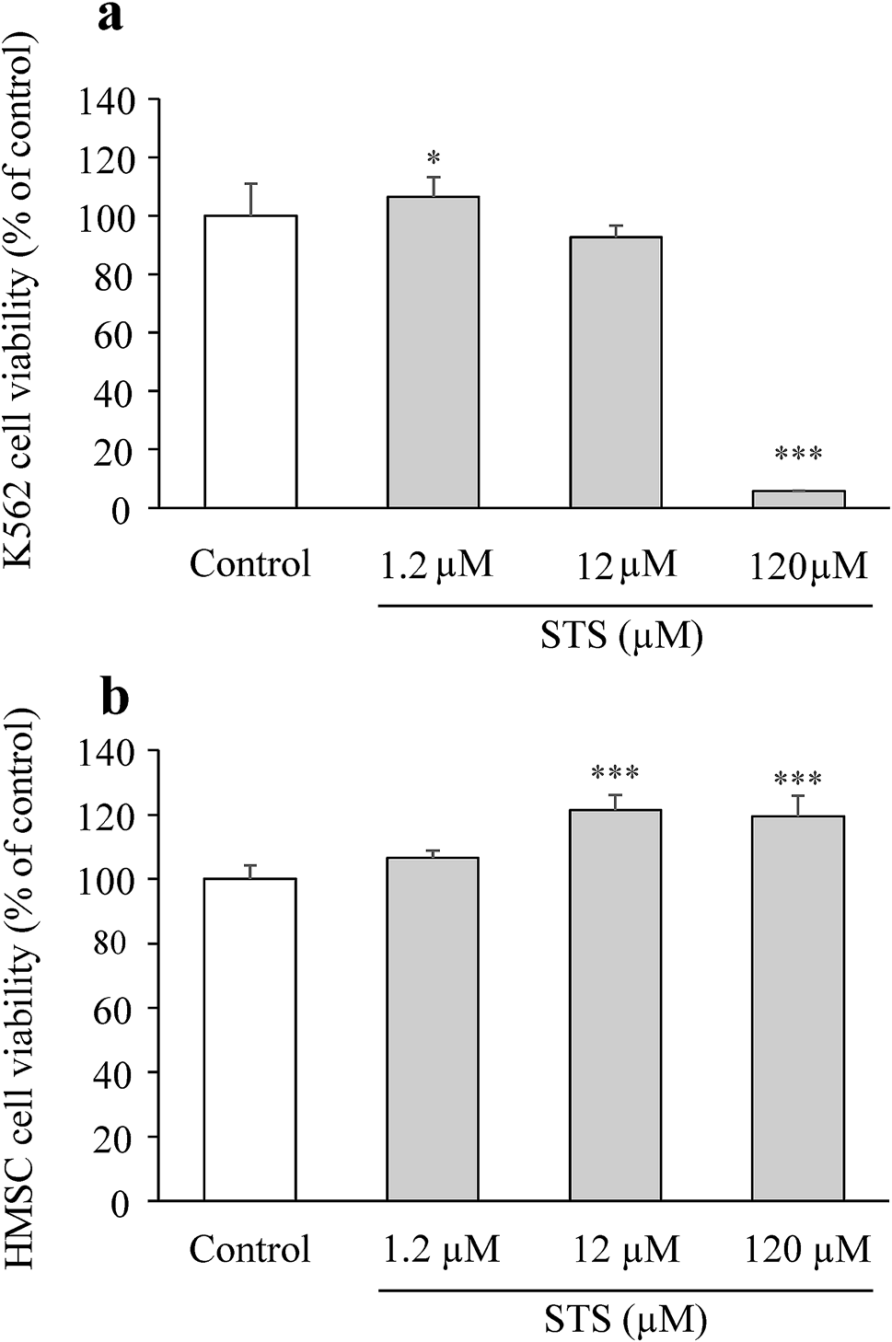
Cytotoxicity evaluation of STS in K562 and HMSC. **a** Cell viability was determined by MTS staining in K562 cells treated with 1.2, 12, and 120 µM of STS for 48 h. Data are expressed as mean ± SD (*n* = 4). Statistical significance from the one-way ANOVA, Tukey’s post hoc test: *p < 0.05, ***p < 0.001 vs. control group. **b** Cell viability was determined by MTS staining in HMSC cells treated with 1.2, 12, and 120 µM of STS for 48 h. Data are presented as mean ± SD (*n* = 4). Statistical significance from the one-way ANOVA, Tukey’s post hoc test: ***p < 0.001 vs. control group

**Fig. 2:**
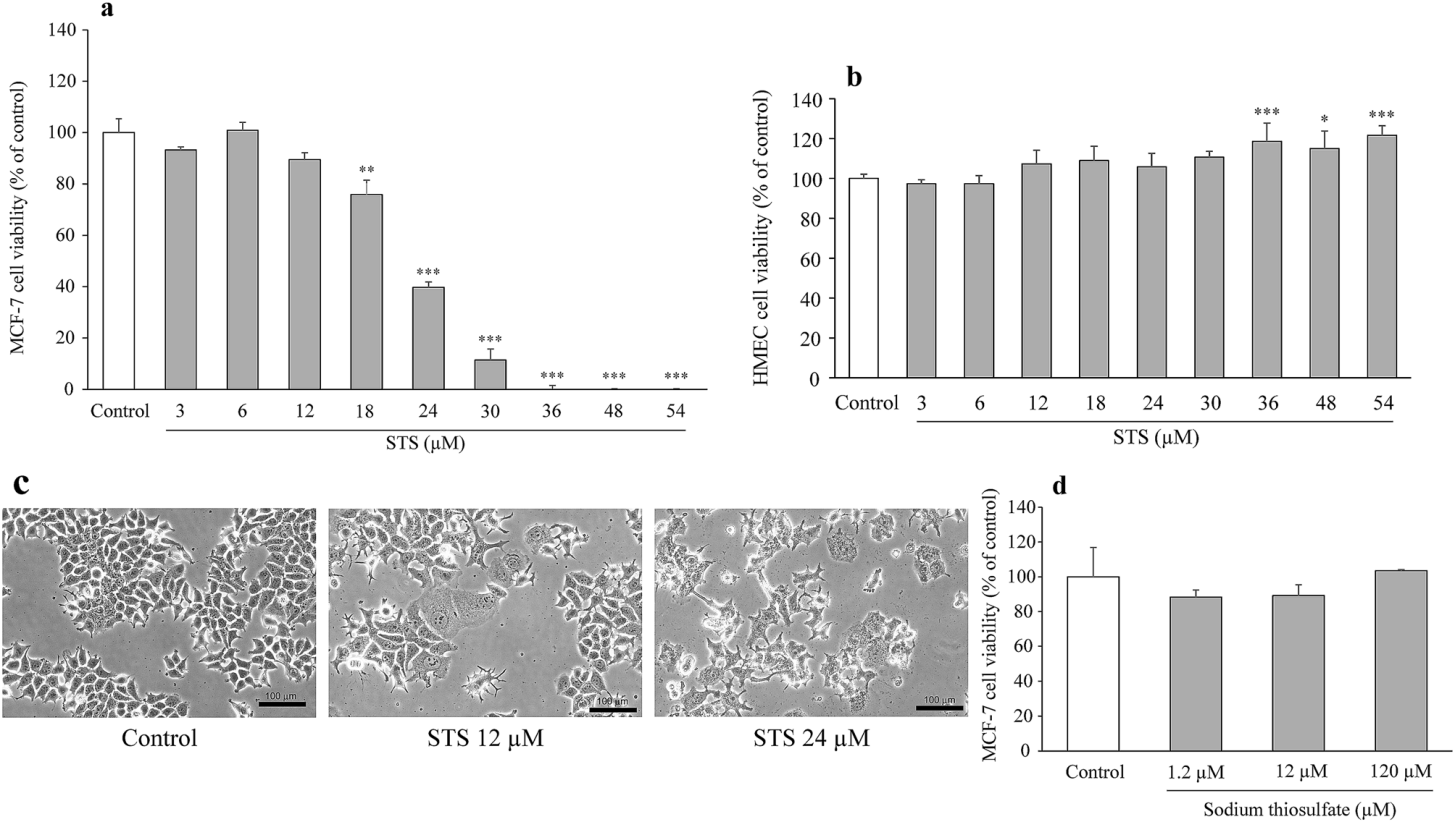
Cytotoxicity evaluation of STS in MCF-7 and HMEC. **a** MCF-7 cells were exposed to STS at various concentrations for 48 h, and then cell viability was detected by MTS assay. Data are expressed as mean ± SD (*n* = 4). Statistical significance from the one-way ANOVA, Tukey’s post hoc test: **p < 0.01, or ***p < 0.001 vs. control group. **b**. HMEC cells were exposed to STS at various concentrations for 48 h, and then cell viability was detected by MTS assay. Data are presented as mean ± SD (*n* = 4). Statistical significance from the one-way ANOVA, Tukey’s post hoc test: *p < 0.05, or ***p < 0.001 vs. control group. **c** Under phase contrast microscopy, morphological changes MCF-7 cells were observed with 12 and 24 µM of STS for 48 h. **d** MCF-7 cells treated with 1.2, 12, and 120 µM of sodium thiosulfate for 48 h. Data are expressed as mean ± SD (*n* = 4). Statistical significance from the one-way ANOVA, Tukey’s post hoc test: *p < 0.05, **p < 0.01, or ***p < 0.001 vs. control group

We conducted a preliminary evaluation of cell viability using several cancer cell lines, and subsequently focused on one floating cell line (K562) and one adherent cell line (MCF-7). STS showed the strongest cytotoxicity in MCF-7 among the cancer cell lines examined, so we employed MCF-7 for further experiments to provide mechanistic insights into the STS’s anticancer effects in vitro. STS did not affect the cell viability of myeloid leukemia cells, K562, at a low STS concentration (12 µM) (*p* = 0.914), but the viability decreased significantly at a high STS concentration (120 µM) (*p* < 0.0001) (*F*_3,12_ = 218.556, *p* < 0.0001) (Fig. 1a), as assessed by one-way ANOVA, followed by Tukey’s post hoc test. In addition, bone marrow cells, which are generally susceptible to anticancer drugs and thus important from the aspect of safety, such as myelosuppression, were also employed for cell viability assay HMSC from bone marrow. As a result, no decrease in the cell viability was observed in HMSC over the STS concentration range used (1.2–120 µM) (*F*_3,12_ = 20.040, *p* < 0.0001), as shown in Fig. 1b, as assessed by one-way ANOVA, followed by Tukey’s post hoc test. A significant decrease in the MCF-7 cell viability (*F*_9,30_ = 259.471, *p* < 0.0001) was observed at 18 µM (*p* = 0.0003) and above, to such an extent that no cell survival was observed at 36 µM (*p* < 0.0001) and above, as shown in Fig. 2a, as assessed by one-way ANOVA, followed by Tukey’s post hoc test. Thus, STS clearly exhibited a concentration-dependent cytotoxicity in MCF-7, with the IC_50_ value being 21.3 ± 2.16 µM. With regard to HMEC, which is a normal mammary epithelial cell line, no decrease in the cell viability was observed even at the high STS concentration of 54 µM (*F*_9,30_ = 7.904, *p* < 0.0001) (Fig. 2b), as assessed by one-way ANOVA, followed by Tukey’s post hoc test. STS-treated cells showed significant morphological changes, such as expansion of cell shapes, cell flattening, loosening of cell–cell contacts, and reduction in the cell population at STS 12 and 24 µM (Fig. 2c). In contrast, the complex ligand, sodium thiosulfate alone, did not affect MCF-7 viability (*F*_3,12_ = 2.699, *p* = 0.093) (Fig. 2d), as assessed by one-way ANOVA, followed by Tukey’s post hoc test.

### Cell cycle analysis

**Fig. 3:**
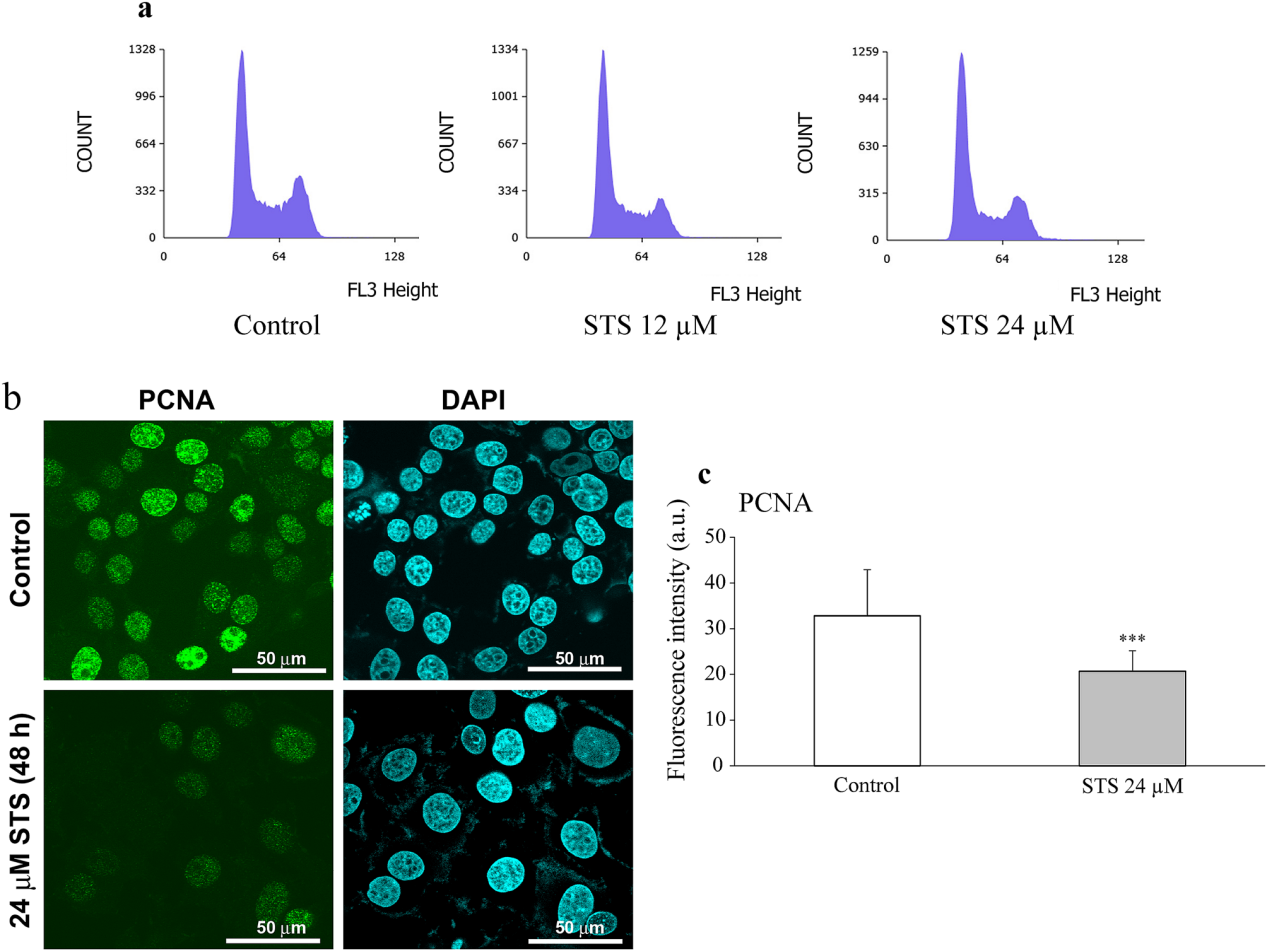
Effects of STS on cell cycle in MCF-7 cells. **a** Representative histogram of MCF-7 cells treated with STS (0, 12 and 24 µM) for 24 h. **b** Representative immunocytochemical images with PCNA antibody of MCF-7 cells after treatment with STS 24 µM for 48 h. **c** Quantification of nuclear expression of PCNA by ImageJ software. The images of 50 cells of each group were analyzed. Data are expressed as mean ± SD (*n* = 50 per group). Statistical significance from Student’s *t*-test: ***p < 0.001

Cell cycle analysis of MCF-7 cells indicated that pretreatment with 12 µM of STS induced cell cycle arrest at the G1 phase (*p* = 0.0014), which led to significant accumulation of cells at the G1 phase (*F*_2,24_ = 9.769, *p* = 0.0008) and decrease of cells at the S phase (*p* = 0.033) (*F*_2,24_ = 5.814, *p* = 0.0087) (Fig. 3a and Table 1). A similar tendency in the cell ratio was observed with an STS concentration of 24 µM (G1 phase: *p* = 0.0040, S phase: *p* = 0.0114) (Table 1), as assessed by one-way ANOVA, followed by Tukey’s post hoc test. To confirm the cell cycle arrest by STS, the immunocytochemical detection for the expression of the proliferation-association proteins, PCNA (Fig. 3c) was performed. PCNA is used for marker of cell proliferation because of its nature to elevate during G1/S phase of cell cycle [17]. As shown in Fig. 3b, c, PCNA expression was significantly lower with STS treatment than with the control (*t*_98_ = 7.7434, *p* < 0.0001), as assessed by Student’s *t* test.

**Table 1:** Effects of STS on cell cycle in MCF-7 cells

Cell population (%)	Control	STS 12 µM	STS 24 µM
G1	45.3 ± 9.77	57.4 ± 2.89^***^	56.2 ± 2.23^***^
S	20.2 ± 3.74	17.0 ± 1.51^***^	16.4 ± 0.78^***^
G2/M	34.2 ± 6.11	25.0 ± 2.34^***^	26.3 ± 1.78^***^

Representative DNA fluorescence of propidium iodide (PI)-stained cells after treatment with STS (12, 24 µM) for 24 h. The data are expressed as mean ± SD (*n* = 9 per group). Statistical significance from the one-way ANOVA, Tukey’s post hoc test: *p < 0.05, **p < 0.01, or ***p < 0.001 vs. control group

*STS* silver thiosulfate

### Evaluation of the mode of cell death

To evaluate the mode of cell death induced by STS, we measured dead cells by PI staining of MCF-7. The percentages of cell death at STS concentrations of 0 µM, 12 µM, and 24 µM were 10.6 ± 4.74%, 23.1 ± 6.46% (*p* = 0.0361), and 53.0 ± 14.1% (*p* < 0.0001), respectively (*F*_2,18_ = 42.942, *p* < 0.0001), as assessed by one-way ANOVA, followed by Tukey’s post hoc test.

**Fig. 4:**
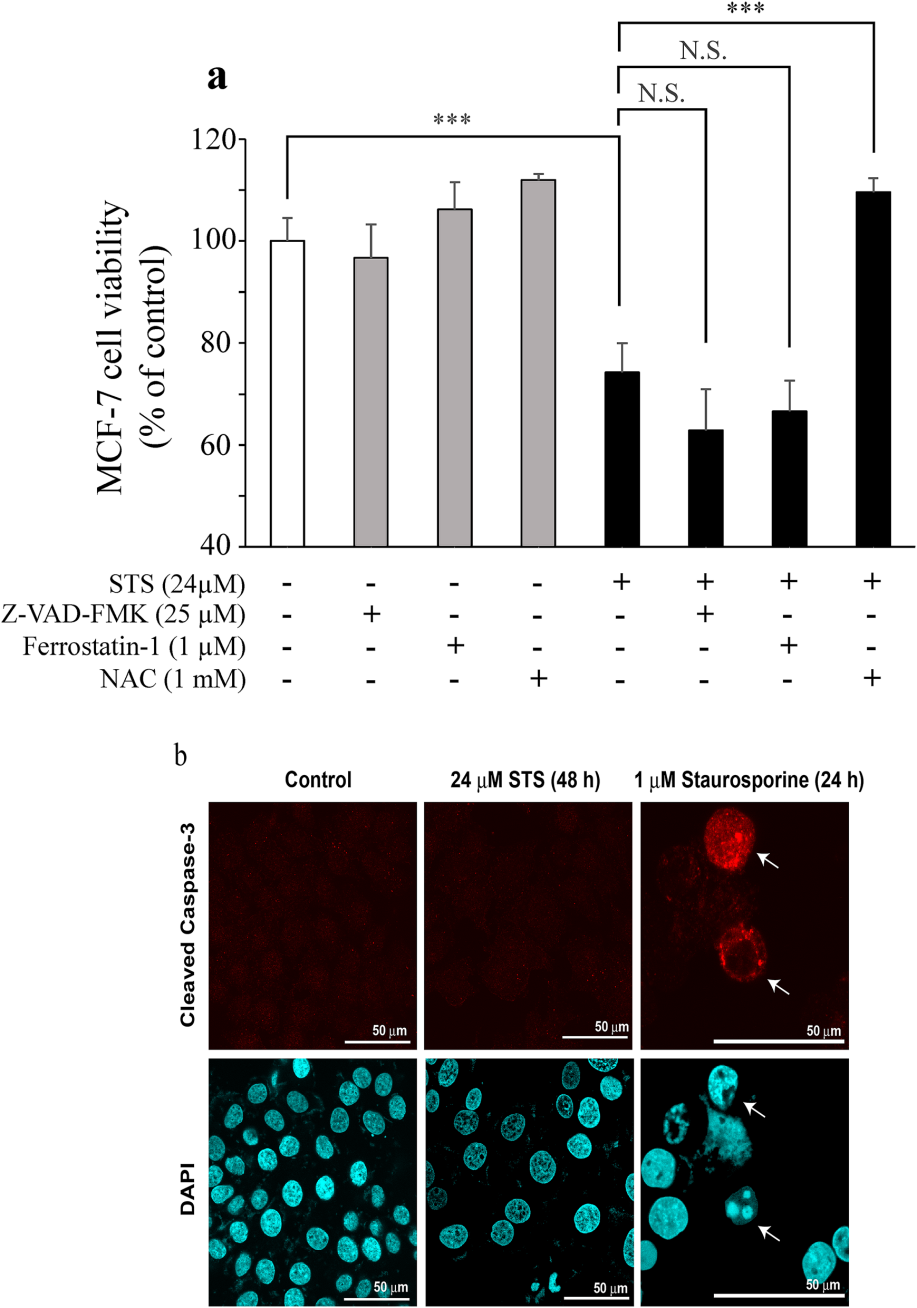
Mode of cell death induced by STS. a MCF-7 cells were pretreated (1 h) with an apoptosis inhibitor (Z-VAD-FMK), a ferroptosis inhibitor (ferrostatin-1), or an antioxidant NAC. Cell viability of MCF-7 cells was measured after treatment with STS (24 µM) for 48 h. Data are presented as mean ± SD (*n* = 4). Statistical significance from two-way ANOVA, Tukey’s post hoc test: ***p < 0.001. **b** Representative immunocytochemical images with cleaved caspase-3 of MCF-7 cells after treatment with STS 24 µM for 48 h. The white arrows indicate cells that have apoptotic cell death after treatment with 1 µM staurosporine for 24 h

**Fig. 5:**
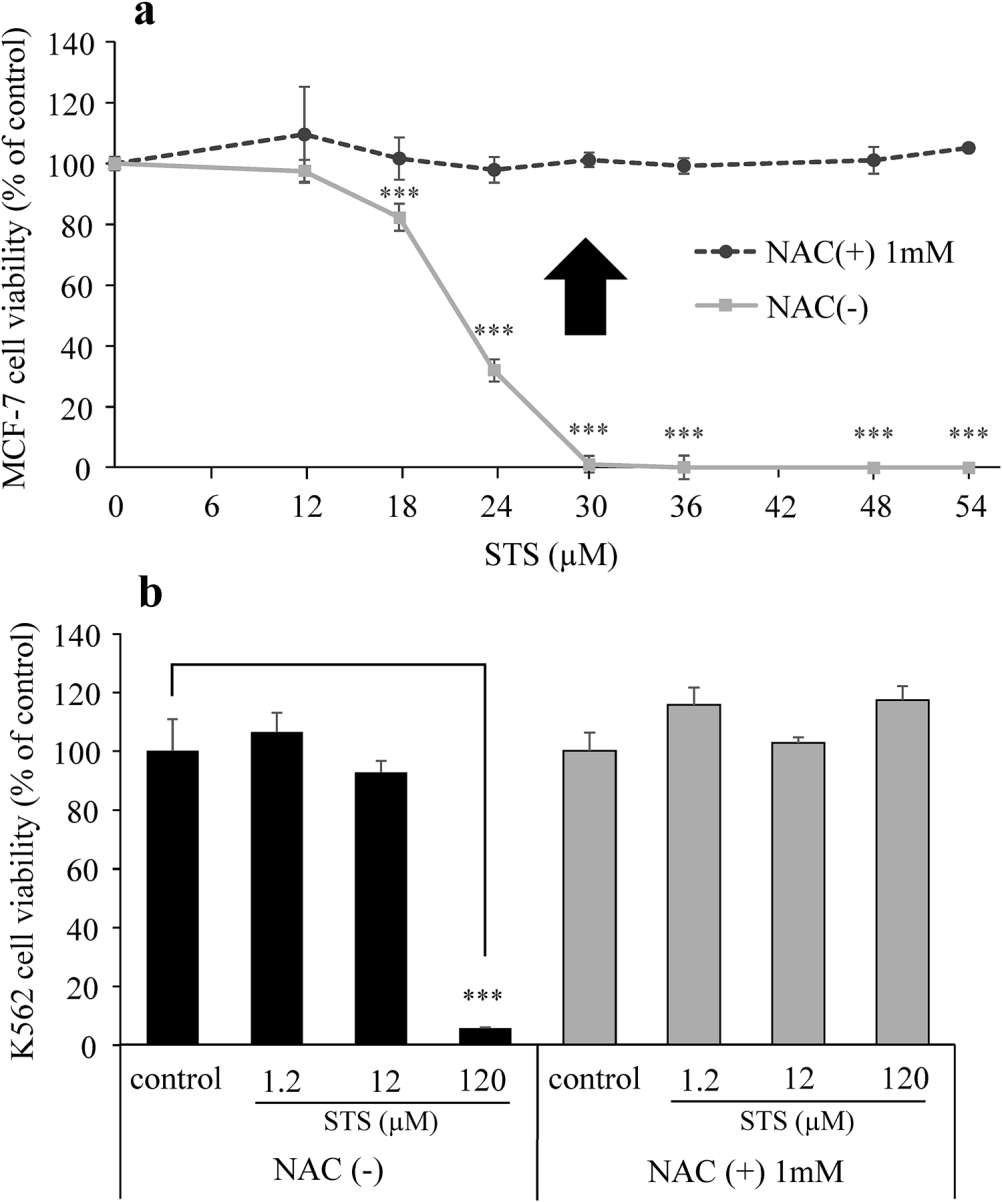
Effects of STS on cell viability in the presence or absence of an antioxidant NAC. **a** MCF-7 cells were pretreated (1 h) with NAC (1 mM) or without it. Cell viability of MCF-7 cells was measured after treatment with STS at various concentrations for 48 h. Data are presented as mean ± SD (*n* = 3). Statistical significance from two-way ANOVA, Tukey’s post hoc test: ***p < 0.001. **b** K562 cells were pretreated (1 h) with NAC (1 mM) or without it. Cell viability of K562 cells was measured after treatment with 1.2, 12, and 120 µM of STS for 48 h. Data are presented as mean ± SD (*n* = 4). Statistical significance from two-way ANOVA, Tukey’s post hoc test: ***p < 0.001

There are various types of cell death, such as apoptosis, necrosis, and ferroptosis. Then, treatment of MCF-7 with Z-VAD-FMK (an apoptosis inhibitor) or ferrostatin-1 (a ferroptosis inhibitor) was performed to evaluate the mode of cell death induced by STS. When MCF-7 cells were pretreated with NAC, which is glutathione precursor and an oxidant scavenger, the cell viability after exposure to 24 µM of STS was as high as that of the control group, as shown in Fig. 4a. Two-way ANOVA revealed a significant effect of STS (STS: *F*_1,24_ = 175.318, *p* < 0.0001), significant effects of inhibitors (*F*_3,24_ = 50.295, *p* < 0.0001), and a significant interaction between STS and inhibitors (*F*_3,24_ = 18.318, *p* < 0.0001) on MCF-7 viability. Tukey’s post hoc analysis showed that NAC significantly reversed the cytotoxic effect of STS (*p* < 0.0001). In brief, no suppression of STS-induced cell death was observed even with these inhibitors, and the cell viabilities remained low. Moreover, to confirm that STS did not induce apoptosis, an immunocytochemical detection for the activation of caspase-3, the apoptosis-related proteins, was performed. As shown in Fig. 4b, the MCF-7 cells treated with staurosporine 1 µM (as a positive control) exhibited an activation of caspase-3, while STS-treated cells did not show an activation of caspase-3. Therefore, it was suggested that the cell death induced by STS was not apoptosis. Furthermore, when MCF-7 cells were exposed to STS at various concentrations after pretreatment with NAC, high cell viabilities were seen at all concentrations examined (Fig. 5a). Two-way ANOVA revealed a significant effect of STS (*F*_7,32_ = 175.658, *p* < 0.0001), significant effects of NAC (*F*_1,32_ = 1538.885, *p* < 0.0001), and a significant interaction between STS and NAC (*F*_7,32_ = 162.350, *p* < 0.0001) on MCF-7 viability. Tukey’s post hoc analysis showed that NAC significantly reversed the cytotoxic effect from 24 µM of STS (*p* < 0.0001). Similarly, as shown Fig. 5b. Two-way ANOVA revealed a significant effect of STS (*F*_3,24_ = 125.794, *p* < 0.0001), significant effects of NAC (*F*_1,24_ = 43.213, *p* < 0.0001), and a significant interaction between STS and NAC (*F*_3,24_ = 175.746, *p* < 0.0001) on K562 viability. Tukey’s post hoc analysis showed that NAC significantly reversed the cytotoxic effect at 120 µM of STS (*p* < 0.0001).

### Measurement of intracellular ROS levels

**Fig. 6:**
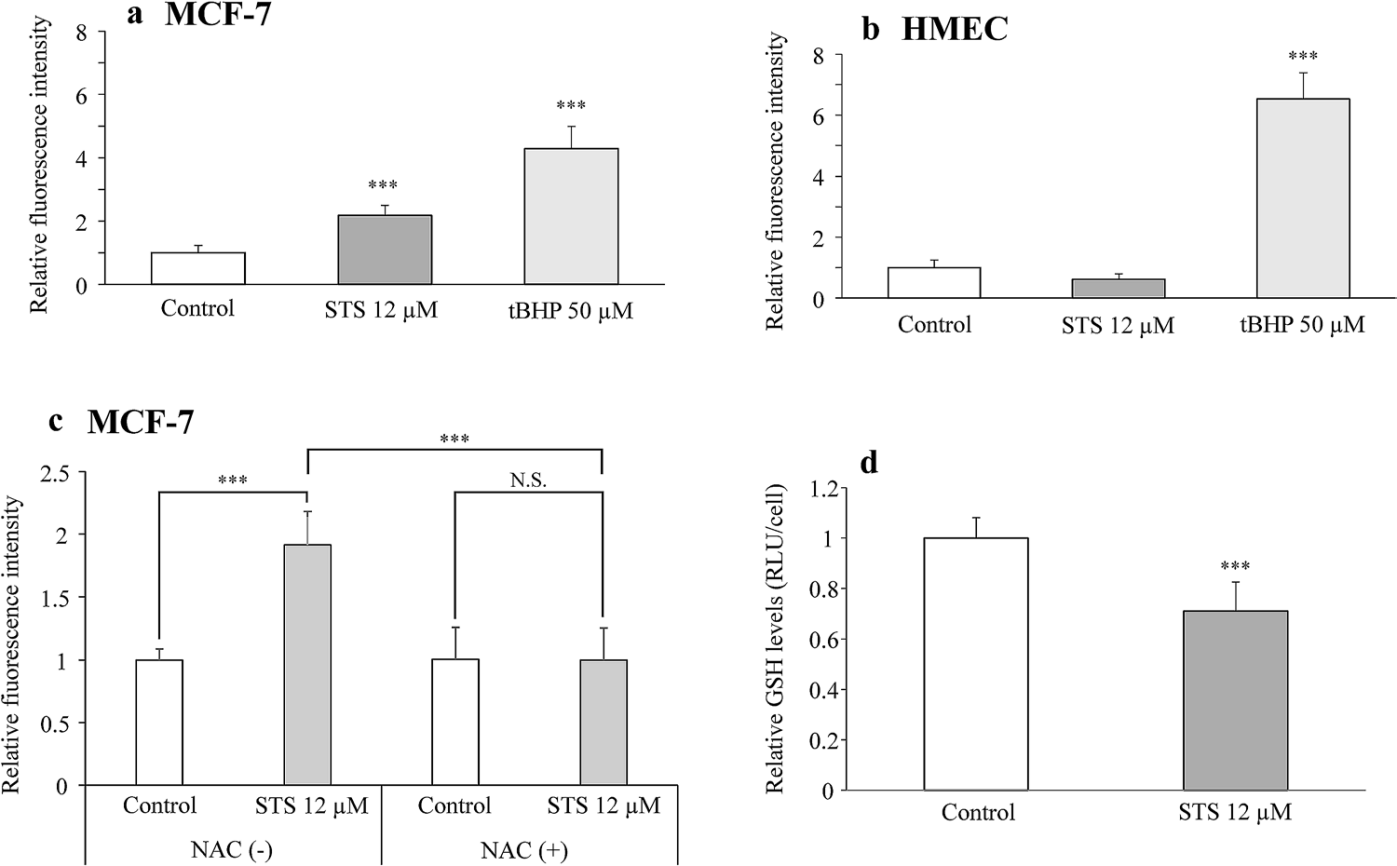
Effects of STS on ROS and GSH levels in MCF-7 and HMEC derived from human mammary epithelial cells. **a** MCF-7 cells were exposed to 12 µM STS for 30 min, or tBHP50 µM as a positive control. ROS levels were detected by flow cytometry-based assay using H2DCFDA. Data are presented as mean ± SD. (control: *n* = 9, STS: *n* = 4, tBHP: *n* = 3). Statistical significance from one-way ANOVA, Dunnett’s test: ***p < 0.001 vs. control group. **b** HMEC cells were exposed to 12 µM STS for 30 min or 50 µM tBHP as a positive control. ROS levels were detected by a flow cytometry-based assay using H2DCFDA. Data are presented as mean ± SD. (control: *n* = 10, STS: *n* = 6, tBHP: *n *= 3). Statistical significance from one-way ANOVA, Tukey’s pot hoc test: ***p < 0.001. **c** MCF-7 cells were pretreated with NAC for 1 h and exposed to 12 µM STS for 30 min. ROS levels were detected by a flow cytometry-based assay using H2DCFDA. Data are expressed as mean ± SD (*n* = 5 per group). Statistical significance from two-way ANOVA, Tukey’s post hoc test: ***p < 0.001. **d** MCF-7 cells were exposed to 12 µM STS for 48 h. GSH levels were measured by luminescence assay. Data are expressed as mean ± SD (*n* = 4 per group). Statistical significance from Student’s *t*-test: ***p < 0.001

Since pretreatment with NAC completely suppressed the STS-induced cell death (Fig. 5a), ROS was considered to be involved in the cell death induced by STS; thus, we subsequently measured the intracellular ROS levels. After treatment with 12 µM of STS, the intracellular ROS level in MCF-7 cells elevated to approximately twice (*p* = 0.0004) that of the control group (*F*_2,13_ = 90.861, *p* < 0.0001) (Fig. 6a), as assessed by one-way ANOVA, followed by Tukey’s post hoc test. In HMEC, the intracellular ROS level was elevated by treatment with tBHP (*p* < 0.0001), but the intracellular ROS level was not changed (*p* = 0.1442) after exposure to STS (*F*_2,16_ = 296.124, *p* < 0.0001) (Fig. 6b), as assessed by one-way ANOVA, followed by Tukey’s post hoc test. As shown in Fig. 6c, STS (12 µM) increased the intracellular ROS levels significantly as compared with the control group (i.e., no treatment with STS) in the absence of NAC (*p* < 0.0001). In contrast, STS did not cause any accumulation of intracellular ROS (*p* = 1.0000) in the presence of NAC, indicating that the presence of NAC abolished STS-induced increase in ROS by STS (*p* < 0.0001). Two-way ANOVA revealed a significant effect of STS (*F*_1,16_ = 19.703, *p* = 0.0004), significant effects of NAC (*F*_1,16_ = 19.895, *p* = 0.0004), and a significant interaction between STS and NAC (*F*_1,16_ = 20.271, *p* = 0.0004) on MCF-7 intracellular ROS levels. Tukey’s post hoc analysis showed that NAC significantly reversed the accumulated ROS levels induced by 12 µM of STS (*p* < 0.0001).

### Measurement of intracellular glutathione levels

To investigate the mechanisms of the intracellular ROS accumulation induced by STS, intracellular levels of GSH, which are one of the major intracellular antioxidants, were measured. The GSH levels (as measured by luminescence assay) in MCF-7 cells treated with STS 12 µM for 48 h were significantly reduced (*t*_6_ = 4.1002, *p* = 0.0064) as compared with the untreated control cells (Fig. 6d), as assessed by Student’s *t* test.

## Discussion

Since STS consists of silver (as a metal) and sodium thiosulfate (as a complex ligand), both of which have been used for medical purposes, the clinical use of STS was considered to be safe. In this study, we aimed to investigate the anticancer activity of STS and its cancer cell selectivity and performed cell viability assay, cell cycle analysis, dead cell assay, morphological inspection, and ROS measurement to clarify its mechanisms.

STS showed a concentration-dependent cytotoxicity in K562 and MCF-7 cells (Figs. 1a and 2a). STS showed a potent cytotoxic effect on MCF-7 around IC_50_ (21.3 µM) (Fig. 2a). In addition, STS exposure exhibited significant morphological changes (Fig. 2c), indicating the cytotoxicity of STS on a visual basis. Some studies have reported that the anticancer effect of silver complexes is not due to ligands but due to the silver element itself [18], which was also supported by the results of the present research (i.e., no cytotoxicity with sodium thiosulfate, which is the ligand of STS, as shown in Fig. 3d). The effects of other silver complexes on the viability of MCF-7 cells have been elucidated in a few reports. The amino-NHC complexes of Ag showed cytotoxicity to MCF-7 with an IC_50_ of 28.68 ± 0.69 µM [19]. STS showed a similar anticancer effect as that of this silver complex. In addition, according to the studies of phosphine silver thiocyanate compounds, NHC carbene silver(I) complexes, and binuclear Silver(I)-NHC carbene complexes, the IC_50_ of the cell viability of MCF-7 was found to be in the range of 3.00–3.59 µM, 1.15–6.17 µM, and 1.48–4.71 µM, respectively [18, 20, 21]. The anticancer effect of STS on MCF-7 was found to be somewhat weaker than that of these silver complexes.

On the other hand, the cancer-cell selectivity of these silver complexes has not been confirmed so far. With regard to binuclear silver(I)-NHC carbene complexes, no cancer cell selectivity was observed between MCF-7 and 3Y3-L1 (normal mouse fetal fibroblasts cells) [21]. When comparing MCF-7 with L-929 (normal cells from the adipose tissue of mice) [20], cancer cell selectivity was observed, but there was no significant difference in their IC_50_ values. Moreover, Guarra and coworkers evaluated the cell selectivity of the silver complex with a fluorescent anthracenyl-functionalized benzimidazole ligand in various human cancer cells and normal cells, and they reported that the cancer cell selectivity of the silver complex was as low as that of cisplatin [22]. In the present study, we performed cell viability assays using two types of normal human cells, i.e., HMSC and HMEC. In both these cells, no cytotoxicity was observed even at higher concentrations of STS, while STS showed anticancer effects in MCF-7 cells with an IC_50_ of 21.3 µM. A comparative study on the viability of MCF-7 and HMEC, which are the same mammary epithelial cells, was considered experimentally appropriate for showing cancer cell selectivity. In addition, regarding bone marrow suppression that frequently occurs as an adverse effect of anticancer drugs, we examined cytotoxicity to HMSC. As a result, STS did not affect the cell viability of HMSC even at a high concentration of 120 µM, and no myeloid cytotoxicity to normal human bone marrow cells was observed with STS (Fig. 1b), although STS significantly reduced the cell viability in K562 myeloid leukemia cells. These results suggest that STS exhibits cancer cell-selective cytotoxicity, being unlikely to cause myelosuppression.

Since MCF-7 exposed to STS was stained with PI, it was clarified that STS induced cell death in MCF-7 cells. In addition, the cell cycle analysis showed that the cell population at the G1 phase significantly increased (Table 1 and Fig. 3a), suggesting that STS arrests the cell cycle of MCF-7 at the G1 phase. The decrease in the expression of PCNA, which is a marker of cell proliferation, indicated that the proliferation of MCF-7 is suppressed by STS (Fig. 3b, c). Regarding the mode of cell death, Z-VAD-FMK, and ferrostatin-1 could not suppress the STS-induced cell death induced by STS (Fig. 4a). It was thus considered that the mode of the cell death caused by STS could be different from apoptosis and ferroptosis. Non-activation of caspase-3 (Fig. 4b) supported our findings that STS did not induce apoptosis (as shown in Fig. 4a) in MCF-7. Moreover, the concentration-dependent anticancer effect of STS on MCF-7 cells was completely suppressed by pretreatment with NAC (Fig. 5a). The measurement of ROS levels in this study clarified that STS led to accumulation of intracellular ROS in MCF-7 (Fig. 6a), but not in HMEC (Fig. 6b). Since the ROS accumulation in MCF-7 was abolished by pretreatment with NAC, it was indicated that the main cause of cell death and cell cycle arrest at the G1 phase induced by STS was due to the cancer cell-selective accumulation of ROS. ROS is generally involved in promoting the growth of cancer cells and malignant cancers, increasing the mutation rate, and causing the development of cancer-specific genetic abnormalities [23]. Elevated ROS levels are observed with platinum anticancer drugs, such as cisplatin [24] and for other silver complexes [8, 9]. Chao and coworkers reported that, when oxidative stress progresses beyond the control of cell-protective mechanisms, excessive accumulation of ROS can cause damage to various cellular components, such as lipids, proteins, mitochondria, and DNA, finally causing cell death [9].

Cell viability assay showed that STS has cancer cell selectivity in terms of cytotoxic effects, but the underlying mechanism remains unclear. In this study, ROS accumulation was not observed in HMEC, which was not affected by STS. Elevated ROS levels induce cell death [25]. Trachootham et al*.* reported that intracellular ROS and antioxidants were elevated in transformed cells. They demonstrated that an inhibitor of the glutathione antioxidant system induced cell death of the transformed cells. Inhibition of the antioxidant system resulted in excessive accumulation of ROS to a threshold that triggers cell death [26]. Thus, we evaluated whether STS affected intracellular GSH levels or not. As a result, the reduction in the intracellular GSH levels was observed after STS treatment using MCF-7 (Fig. 6d). NAC suppressed the STS-induced ROS accumulation and cell death completely (Fig. 6c), suggesting that reduction of GSH could be involved in the ROS accumulation induced by STS. Cancer cells are generally susceptible to changes in ROS levels because they have abundant ROS and antioxidants [23]. When STS reduce the GSH levels of antioxidants, the intracellular ROS levels in cancer cells would increase and exceed the threshold, consequently causing cell death. It is considered that the selective cell death in the cancer cell lines observed in this study may be due to the large difference in the levels of ROS and antioxidants in cancer cells and the accumulation of ROS exceeding the threshold value. On the other hand, the difference in the levels of ROS and antioxidants is known to be small in normal cells, which may be the reason why no ROS accumulation was observed in HMEC.

There are some limitations of this study. We evaluated the anticancer effect of STS mainly using MCF-7, but different mechanisms (other than ROS accumulation) related with intracellular GSH reduction might be involved in STS actions for other cell lines. Detailed molecular mechanisms of STS actions remain unclear and should be further clarified. This study was conducted under in vitro conditions; therefore, in vivo effects of STS remain unknown.

This study revealed, for the first time, that STS leads to accumulation of ROS in cells and causes cell death, thereby exhibiting cancer cell-selective cytotoxicity in vitro. The results of the present study warrant further investigation on the detailed mechanism of STS actions, as well as its in vivo effectiveness and safety for clinical application.

## Abbreviations

ANOVA: Analysis of variance
BSA: Bovine serum albumin
DAPI: 4′,6-Diamidino-2-phenylindole
DMEM: Dulbecco’s modified Eagle’s medium
DPBS: Dulbecco’s phosphate-buffered saline
FBS: Fetal bovine serum
GSH: Glutathione
H2DCFDA: 2′,7′-Dichlorodihydrofluorescein diacetate
HMEC: Human mammary epithelial cells
HMSC: Human mesenchymal stem cells
MTS: (3-(4,5-Dimethylthiazol-2-yl)-5-(3-carboxymethoxyphenyl)-2-(4-sulfophenyl)-2H-tetrazolium)
NAC: *N*-Acetylcysteine
NHC: *N*-Heterocyclic carbene
PCNA: Proliferating cell nuclear antigen
PI: Propidium iodide
RNase A: Ribonuclease A
ROS: Reactive oxygen species
RPMI-1640: Roswell park memorial institute-1640
STS: Silver thiosulfate complex
tBHP: Tert-Butyl hydroperoxide

## References

[1] Oun R, Moussa YE, Wheate NJ (2018). The side effects of platinum-based chemotherapy drugs: a review for chemists. Dalton Trans.

[2] Ndagi U, Mhlongo N, Soliman ME (2017). Metal complexes in cancer therapy—an update from drug design perspective. Drug Des Dev Ther.

[3] Tan SJ, Yan YK, Lee PP, Lim KH (2010). Copper, gold and silver compounds as potential new anti-tumor metallodrugs. Future Med Chem.

[4] Kuehl R, Brunetto PS, Woischnig AK, Varisco M, Rajacic Z, Vosbeck J (2016). Preventing implant-associated infections by silver coating. Antimicrob Agents Chemother.

[5] Innes ME, Umraw N, Fish JS, Gomez M, Cartotto RC (2001). The use of silver coated dressings on donor site wounds: a prospective, controlled matched pair study. Burns.

[6] Munster AM, Helvig E, Rowland S (1980). Cerium nitrate–silver sulfadiazine cream in the treatment of burns: a prospective evaluation. Surgery.

[7] Nowack B, Krug HF, Height M (2011). 120 years of nanosilver history: implications for policy makers. Environ Sci Technol.

[8] Engelbrecht Z, Meijboom R, Cronjé MJ (2018). The ability of silver(I) thiocyanate 4-methoxyphenyl phosphine to induce apoptotic cell death in esophageal cancer cells is correlated to mitochondrial perturbations. Biometals.

[9] Chen C, Zhou L, Xie B, Wang Y, Ren L, Chen X (2020). Novel fast-acting pyrazole/pyridine-functionalized *N*-heterocyclic carbene silver complexes assembled with nanoparticles show enhanced safety and efficacy as anticancer therapeutics. Dalton Trans.

[10] Fichtner I, Cinatl J, Michaelis M, Sanders LC, Hilger R, Kennedy BN (2012). *In vitro* and *in vivo* investigations into the carbene silver acetate anticancer drug candidate SBC1. Lett Drug Des Discov.

[11] Chen X, Yang Q, Chen J, Zhang P, Huang Q, Zhang X (2018). Inhibition of proteasomal deubiquitinase by silver complex induces apoptosis in non-small cell lung cancer cells. Cell Physiol Biochem.

[12] Tomioka T (1999). Antimicrobial agent composed of silica-gel with silver complex. Inorg Mater.

[13] Zakharov S, Vaneckova M, Seidl Z, Diblik P, Kuthan P, Urban P (2015). Successful use of hydroxocobalamin and sodium thiosulfate in acute cyanide poisoning: a case report with follow-up. Basic Clin Pharmacol Toxicol.

[14] Goel R, Cleary SM, Horton C, Kirmani S, Abramson I, Kelly C (1989). Effect of sodium thiosulfate on the pharmacokinetics and toxicity of cisplatin. J Natl Cancer Inst.

[15] Freyer DR, Chen L, Krailo MD, Knight K, Villaluna D, Bliss B (2017). Effects of sodium thiosulfate versus observation on development of cisplatin-induced hearing loss in children with cancer (ACCL0431): a multicentre, randomised, controlled, open-label, phase 3 trial. Lancet Oncol.

[16] Ishikawa F, Ushida K, Mori K, Shibanuma M (2015). Loss of anchorage primarily induces non-apoptotic cell death in a human mammary epithelial cell line under atypical focal adhesion kinase signaling. Cell Death Dis.

[17] Bologna-Molina R, Mosqueda-Taylor A, Molina-Frechero N, Mori-Estevez AD, Sánchez-Acuña G (2013). Comparison of the value of PCNA and Ki-67 as markers of cell proliferation in ameloblastic tumors. Med Oral Patol Oral Cir Bucal.

[18] Ferreira E, Munyaneza A, Omondi B, Meijboom R, Cronjé MJ (2015). The effect of 1:2 Ag(I) thiocyanate complexes in MCF-7 breast cancer cells. Biometals.

[19] Wang CH, Shih WC, Chang HC, Kuo YY, Hung WC, Ong TG (2011). Preparation and characterization of amino-linked heterocyclic carbene palladium, gold, and silver complexes and their use as anticancer agents that act by triggering apoptotic cell death. J Med Chem.

[20] Şahin N, Şahin-Bölükbaşı S, Tahir MN, Arıcı C, Çevik E, Gürbüz N (2019). Synthesis, characterization and anticancer activity of allyl substituted *N*-Heterocyclic carbene silver(I) complexes. J Mol Struct.

[21] Asif M, Iqbal MA, Hussein MA, Oon CE, Haque RA, Khadeer Ahamed MB (2016). Human colon cancer targeted pro-apoptotic, anti-metastatic and cytostatic effects of binuclear silver(I)–N-Heterocyclic carbene (NHC) complexes. Eur J Med Chem.

[22] Guarra F, Busto N, Guerri A, Marchetti L, Marzo T, García B (2020). Cytotoxic Ag(I) and Au(I) NHC-carbenes bind DNA and show TrxR inhibition. J Inorg Biochem.

[23] Sullivan LB, Chandel NS (2014). Mitochondrial reactive oxygen species and cancer. Cancer Metab.

[24] Chen B, Shen Z, Wu D, Xie X, Xu X, Lv L, et al. Glutathione peroxidase 1 promotes NSCLC resistance to cisplatin via ROS-induced activation of PI3K/AKT pathway. BioMed Res Int. 2019; ID 7640547. 10.1155/2019/7640547 PMC6457285 31032363

[25] Ryter SW, Kim HP, Hoetzel A, Park JW, Nakahira K, Wang X (2007). Mechanisms of cell death in oxidative stress. Antioxid Redox Signal.

[26] Trachootham D, Alexandre J, Huang P (2009). Targeting cancer cells by ROS-mediated mechanisms: a radical therapeutic approach?. Nat Rev Drug Discov.

